# Supplementary material for: Ileocolic Intussusception Due to Low-Grade Appendiceal Mucinous Neoplasm
Source: Diagnostics (Basel). 2024 Sep 14;14(18):2040. doi: 10.3390/diagnostics14182040 (PMC11431097; doi:10.3390/diagnostics14182040)
Supplement: Supplementary file 1 [file diagnostics-14-02040-s001.zip › diagnostics-3172591-supplementary.pdf]

Table S1: Cases with appendiceal LAMN and intussusception reported between January 2014 and August 2024.

Authors	Year	Gender	Age	Initial presentation	Pathology	Mucocele size (cm)	CT findings of mucocele	Intussusception appearance	Preoperative imaging study	Operation
Sun et al[9]	2014	F	57	hematochezia, changes in defecation habits, mild swelling and pain in the right lower quadrant of abdomen	LAMN pTis and rectal medium-low differentiated adenocarcinoma (cT4N1M0)	not reported	cystic	Appendix intussuscepted into the cecum	CT, colonoscopy, biopsy	Resection of both the appendix and ileocecum, radical resection and regular chemotherapy for rectal carcinoma
Chan et al[10]	2014	F	41	intermittent, right-sided abdominal pain, abdominal distension, nausea, and vomiting	LAMN pTis	not reported	cystic	Ileocolic intussusception	CT, Colonoscopy	Laparoscopic right hemicolectomy
Chua et al[11]	2016	F	30	worsening right lower abdominal pain associated with nausea, vomiting, and diarrhea for 2 days	LAMN pTisN0	not reported	cystic	ileocolic intussusception to transverse colon	CT	Stapled partial caecectomy with appendicectomy
Nakamatsu et al[12]	2018	F	43	right lower quadrant pain for several days	LAMN pTis	4	cystic	Ileocolic intussusception to transverse colon	CT, colonoscopy, biopsy	Elective laparoscopy assisted ileocecal resection with lymph node dissection
Houlzé-Laroye et al[14]	2019	F	35	abdominal symptoms evoking an intestinal obstruction	LAMN	not reported	cystic with mural calcification	Ileocolic intussusception to transverse colon	CT	Exploratory laparotomy and bowel resection

Davey et al[13]	2020	M	42	sudden onset abdominal pain and bloody diarrhea.	LAMN pTisN0	8.6	cystic	ileocolic intussusception to hepatic flexure	CT, Colonoscopy	laparotomy and right hemicolectomy
Teke et al[5]	2020	F	37	acute onset, right lower abdominal pain	LAMN pTis	5.3	cystic	Appendix intussuscepting into the caecum	CT	Ileocaecal resection
Moniakis et al[15]	2021	F	65	vomiting, exacerbation of abdominal pain, and electrolyte disorders	LAMN pTisN0 + 6cm terminal ileal inflammatory fibroid polyp	not reported	not found	ileocecal intussusception	CT, Colonoscopy	right hemicolectomy
Yang et al[16]	2022	M	47	paroxysmal abdominal pain and postprandial bloating for 3 days	LAMN & intestinal hemangioma	5	not found	ileocecal intussusception	CT	laparotomy and right hemicolectomy
Lin et al[17]	2022	F	58	Intermittent episodes of epigastric pain with periumbilical tenderness.	LAMN pTis	5	cystic with mural calcification	ileocolic intussusception to transverse colon	CT, Colonoscopy	Laparoscopic right hemicolectomy
Harvitkar et al[18]	2022	M	76	dull pain in the lower abdomen and a palpable lump in the right lower abdomen.	LAMN	not reported	not found	ileocolic intussusception	CT	Laparoscopic quarter colectomy with ileoascending anastomosis
Liapis et al[22]	2023	F	80	progressively worsening diffuse abdominal pain for the past 24 h	LAMN pT3N0M0	10.5	cystic with hydro-aerial level	Ileocecal intussusception	CT, US	right hemicolectomy

Núñez-Rocha et al[19]	2023	F	32	colicky abdominal pain in the right upper quadrant for 5 days	LAMN pTis	not reported	cystic	ileocolic intussusception to transverse colon	CT	laparoscopic right colectomy
Maloku et al[20]	2023	F	42	severe abdominal pain located under the left costal margin in recent 3 days	LAMN	8	cystic	ileocolic intussusception to transverse colon	CT, MRI	right hemicolectomy
Vale et al[21]	2024	F	54	progressive worsening abdominal distension and diffuse pain more intense in the right lower quadrant and palpable RLQ mass for 4 days	LAMN pTis	3.5	cystic with mural calcification	ileocolic intussusception	US, CT	laparotomy and right hemicolectomy

Abbreviations: F, female; M, male.
